# Effects of floral display size, local open raceme density, patch size, and distance between patches on pollinator behaviour in *Salvia nipponica*

**DOI:** 10.1038/s41598-024-51327-w

**Published:** 2024-01-10

**Authors:** Noriko Murakoshi, Tomoyuki Itagaki, Michio Oguro, Satoki Sakai

**Affiliations:** 1 Graduate School of Life Sciences, Tohoku University, Sendai, Japan; 2 Forestry and Forest Products Research Institute, Tsukuba, Japan

**Keywords:** Behavioural ecology, Evolutionary ecology

## Abstract

Flowers cluster at various spatial scales, so pollinators use information from multiple scales when foraging in natural plant populations. Little is known about the effects of interactions between scales or their relative strength. We examined bumblebee foraging behaviour in a natural population of *Salvia nipponica* in 10 and 7 patches in 2019 and 2020, respectively. We recorded within-patch factors (display size of racemes and local open raceme densities) and patch-level factors (patch size and distance from the nearest patch) and analysed their relationships with pollinator behaviour. The numbers of visits per raceme and flower were mainly affected by the interaction of patch size and raceme density; they were higher in locations with lower raceme density in larger patches. The ratio of flowers visited to all open flowers in a raceme during a raceme visit, which relates to a bumblebee’s choice to leave a raceme, was mainly affected by the interaction of display size and local open raceme density; in 2019 it was higher in racemes with smaller display sizes, while in 2020 the strength and direction of the relationship depended on the open raceme density. These results suggest that pollinators relied on the sizes of flower clusters at different spatial scales when visiting and leaving racemes and adjusted their responses to the sizes of flower clusters depending on the distances between clusters. Therefore, it is important to evaluate factors at various spatial scales and their interactions to fully understand pollinator behaviour in natural plant populations.

## Introduction

Pollinator foraging behaviour is important for fitness of pollinators and animal-pollinated plants and hence many studies have examined factors affecting foraging behaviour^1–4^. These factors include floral display size (number of simultaneously opening flowers on a plant or an inflorescence)^1,4–9^, local plant (or inflorescence) density around a focal plant^4,6,7,10–13^, patch size (size of plant clusters within populations, where a population is defined such that the distances between populations are long and the populations are almost isolated from each other in terms of pollination)^9–11^, and distance between patches^2,14–17^. Using signals from these factors, pollinators may make decisions about visiting and leaving plants and patches. Optimal foraging theory^18^ is based on cost/benefit analyses where obtaining resources such as nectar is a benefit and energy spent foraging is a cost; and it predicts that pollinators should visit and leave plants as to optimize the benefit/cost ratio^19,20^.

Understanding how pollinators evaluate costs and benefits would increase our understanding of how pollinators decide to visit and then leave a flower, plant, or patch. Previous studies have examined the effects of individual signals on pollinator behaviour. (1) Floral display size and patch size: in general, pollinator visitation rate to a plant (or an inflorescence) increases with increasing floral display size^1,4–9^, possibly because larger patches provide more rewards. Similarly, the visitation rate to a patch^2,9,14^ and the residence time (the length of time a pollinator remains in a patch)^16^ increase with increasing patch size. However, the proportion of visited flowers decreases with increasing inflorescence or patch size^2–4^, possibly because of the cost of revisiting already-visited flowers^21^. (2) Local plant density: higher plant density should decrease cost by decreasing distance between plants. In general, pollinators visit more flowers on a plant in sparse areas^4,6,7,10,22,23^. On the other hand, the visitation rate per plant is higher in dense areas, potentially increasing benefits at higher plant density^4,6,7,11–13,24–28^. (3) Distance between patches: lastly, increasing distance between patches increases foraging time and cost of movement when pollinators move between patches^20^.

In natural plant populations, flowers can be grouped at various spatial scales; on inflorescences or racemes within a plant; on a plant cluster within a patch; or in patches with multiple plants. In such heterogenous conditions, pollinators may simultaneously evaluate multiple signals and may assign different levels of importance to different signals. Moreover, different signals may interact in how they affect pollinators. This could be interpreted as pollinators using interactive information obtained from multiple signals. For example, pollinators tend to visit more flowers on larger inflorescences or patches although they tend to visit a smaller proportion of the available flowers^10,19^. This behaviour led to the concept of ideal free distribution (IFD) of pollinators, i.e., a constant visitation rate on each flower irrespective of the size of flower clusters^2,19^. The key assumptions of the IFD are that the cost of movement is negligible, and foragers are omniscient about resource distributions. If these assumptions are violated, distribution of pollinators is predicted to deviate from the IFD^20,29,30^. The cost of movement and the perception of resource distribution may depend on the distance between clusters of resources^20,30^. Therefore, the size of flower clusters (e.g., floral display and patch size) and the distance between them (e.g., local density and distance between patches) may interact in affecting pollinator behaviour, although no interaction between the effects of patch size and distance between patches on pollinator behaviour was observed in a previous study^2^. Moreover, because the cost of movement would depend on the spatial scale, local density within a patch and distance between patches may also interact in affecting pollinator visitation rates.

Previous studies have studied the effects of single factors or only a few factors on pollinator behaviour: population (not patch) sizes and average plant densities in the populations^31–36^, local plant density (density of flowering plants around a focal plant)^1,6–8,12,13,23,25–27,37–45^, or patch size^2,16,46^. Dauber et al.^47^ focused on both patch density and size, but the effects of distance between patches and display sizes of individual plants were not examined. Mustajärvi et al.^28^ studied pollinator behaviour in artificial patches that included two levels of density and size, but studies with more variation in density and size of patches are required because pollinator decision making may become more complicated in more complex foraging sites, possibly including non-linear interactive effects of density and size. In addition, though the effects of distance between patches have been examined^15,17,48^, interactions of distance between patches with other patch characteristics are poorly understood (but see Fragoso et al.^14^, which showed that bees used both patch size and distance between patches when selecting a patch).

The purpose of this study is to determine the interactive effects and relative importance of flower clusters in different spatial scales on pollinator behaviour. Therefore, we examined bumblebee foraging behaviour in a natural population of *Salvia nipponica*. We focused on four factors representing cost and benefit for pollinators: local open raceme density (density of racemes having at least one open flower), patch size, patch distance, and floral display size (number of open flowers on a raceme). We then elucidated their effects and relative strengths, including the interactions among them, on three measures of pollinator behaviour: the number of visits to a raceme, the number of visits to a flower, and the proportion of visited flowers in a raceme.

## Materials and methods

### Plant species

*Salvia nipponica* Miq. (Lamiaceae) is a perennial herb that grows on the forest floor in the Japanese mainland. Each plant produces one or a few racemes (inflorescences), and each raceme bears up to 50 flowers. The yellow and lip-shaped flowers are adichogamous (i.e., stamens and pistils of a flower ripen at the same time), and each flower has 4 ovules. This species is self-compatible but needs pollinators for seed production^3^. Most flowers last only one day, and up to 15 flowers open on a raceme on one day. The mean ± SD number of open flowers of each observed raceme was 3.6 ± 1.7. In our study site, flowering occurs from early September through early October. The primary pollinator is *Bombus diversus* Smith. They visit the flowers mainly for nectar, but sometimes collect pollen^3^. Flowers of *S. nipponica* have a special stamen structure which allows only compatibly-sized pollinators (i.e., bumblebees in our study site) to contribute to their pollination^3^. Other visitors (including small bees, bee flies, and hover flies) do not perform effective pollination (though they try to collect nectar or pollen, their bodies fail to touch anthers, stigmas, or both, and therefore we did not examine them in this study. *S. nipponica* also reproduces clonally by tuberous roots or creating new roots from stems touching the ground, although it usually does not spread over long distances via clonal reproduction. Because the underground structure and history of clonal reproduction are unknown, we regarded an aboveground cluster of shoots, which might have several racemes, as a ramet (individual plant).

### Study site and patches

**Figure 1:**
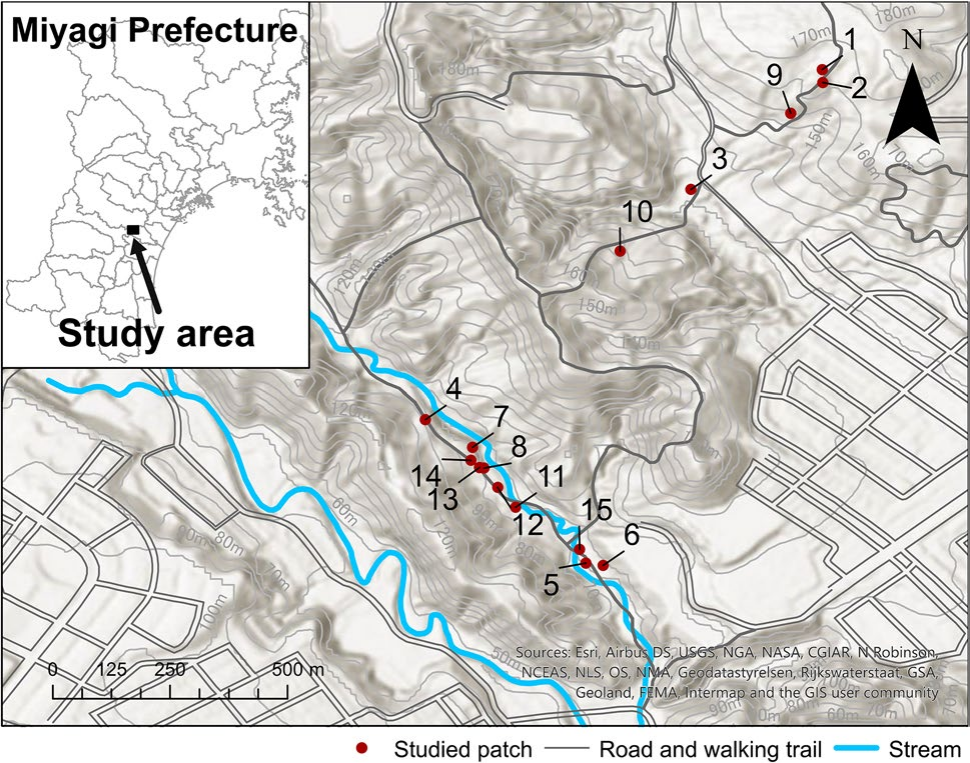
A map of patches studied. The study patches were on flat places or gentle slopes in continuously distributing mixed deciduous and coniferous forests dominated by tree species such as *Abies firma*, *Quercus serrata*, and *Pinus densiflora* or in an adjacent *Cryptomeria japonica* forest. The elevations of the patches were about 80–120 m and the under-canopy vegetation and environmental conditions looked similar. The study site is in two adjacent areas in Kongosawa and Mt. Kagitori National Forests, Miyagi Prefecture, northern Honshu, Japan.

Field work was conducted on the forest floor in two adjacent areas in Kongosawa and Mt. Kagitori National Forests, Miyagi Prefecture, northern Honshu, Japan (Fig. 1). The plant patches selected for this study were on flat areas or gentle slopes in continuously distributed mixed deciduous and coniferous forests dominated by tree species such as *Abies firma*, *Quercus serrata*, and *Pinus densiflora*, or in an adjacent *Cryptomeria japonica* forest. The elevations of the patches were about 80–120 m. Although there were a few other nectar sources for bumblebees, such as *Impatiens textorii* and *Tricyrtis affinis,* around some patches, flowers of *S. nipponica* were much more abundant than those other nectar sources. The under-canopy vegetation and environmental conditions looked similar among the patches. We selected patches of wild *S. nipponica* plants of various sizes. We defined a patch as a group of ramets in which the distances between the ramets were within 5 m because 5 m distances were enough to distinguish patches and would prevent the same genets being assigned to different patches since the species does not spread over long distances. Therefore, the minimum distance between the patches was more than 5 m.

We selected the patches studied as follows. In the middle of September in 2019, we recorded all flowering patches found from the walking trail network running through the study site. We then selected 10 patches so that their patch size distribution showed a wide range and each patch included racemes with multiple display sizes (Table 1; observed display sizes for each patch and year are shown in Supplementary Fig. S1d). We also took care to select patches distributed over a sufficiently small area to allow us to set video cameras and start video recording without large time lags. Many of the patches studied differed between 2019 and 2020 because, in 2020, flowering was early and had ended before we got to the patches or we could no longer find the patches studied in 2019 (Table 1). However, we studied patches nos. 4 and 10 in both years.

**Table 1:** Characteristics of patches studied and the numbers of ramets (a cluster of shoots), racemes, and flowers observed in each patch.

Patch identity	Year studied	Patch area (m^2^)	Patch size (number of ramets in a patch)	Distance to the nearest patch (m)	Number of observed ramets	Number of observed racemes	Number of observed flowers
1	2019	40.61	87	25.28	6	7	18
2	2019	5.24	23	25.82	10	14	49
3	2019	22.66	47	176.47	8	13	29
4	2019	444.93	422	98.54	6	10	21
5	2019	12.19	73	29	7	11	20
6	2019	24.57	80	217.66	3	6	16
7	2019	37.95	173	48.22	3	8	26
8	2019	10.24	88	48.22	8	13	32
9	2019	9.52	84	84.32	7	21	57
10	2019	698.64	538	176.47	13	20	58
4	2020	44.91	111	114.5	9	9	27
10	2020	456.26	971	483	18	18	70
11	2020	4.30	27	51	2	2	7
12	2020	20.83	132	51	7	7	26
13	2020	30.20	154	20.5	9	9	37
14	2020	15.69	64	20.5	8	8	27
15	2020	13.21	59	139	3	3	17

For each patch, we recorded the number of reproductive ramets (patch size). Locations of all the flowering patches were recorded by GPS and planar distances from the nearest patch were calculated for the patches used for the pollinator observations. The distances from the nearest patch were 20.5–483 m (Table 1), which is within the reported range of foraging area for bumblebee pollinators (estimated maximum foraging ranges are at least 449–758 m for four bumblebee species^49^).

### Pollinator observations

Observations of pollinators were carried out on 5 and 7 sunny, warm days between 19 and 25 September 2019 and 16–28 September 2020, respectively (Supplementary Table S1). On each observation day, we selected 1 to 5 patches depending on their flowering conditions and locations within the study site; in particular in 2019, we tried to select northern (no. 1–3, 9, and 10) and southern (no. 4–8) patches on alternate days to minimize the possible effects of the difference in observation dates between the locations, while retaining simultaneous observation of nearby patches for convenience. In each patch, we selected 1–9 ramets and 1–11 racemes, aiming to observe racemes of various display sizes (ranging from 1 to 9 flowers) growing in patches of various sizes. In total, we recorded 5–31 racemes on one day and the total numbers of racemes observed were 123 and 56 in 2019 and 2020, respectively. The total number of ramets, racemes, and flowers used for pollinator observations for each year and patch are shown in Table 1 and the number of observed racemes for each observation day and patch are shown in Supplementary Table S1.

Using 4–9 video cameras (GZ-RX, JVCKENWOOD, Kanagawa, Japan), we recorded pollinator visits to each of the selected racemes for 180 min during 0900-1300, when pollinators were foraging. In both years, a raceme was only observed once. The total observation time was 216 h in 2019 and 168 h in 2020. We observed all open flowers on each selected raceme. Because the flowering phenology and the number of available racemes were different between the two years, 1 to 6 racemes belonging to the same ramets were simultaneously observed by single video cameras in 2019, whereas in 2020, single racemes were observed by single video cameras.

After the field observations, we recorded the number of open flowers (display size) for each selected raceme. A flower was classified as open if its corolla opening was wider than 7 mm, which allows pollinators to enter the flower. Ten racemes dropped one or two flowers during the video recording, so the field-measured numbers of open flowers were later adjusted for each pollinator visit using the video recordings for these racemes. Also, the number of other racemes having open flowers within a 1.5 m radius from the focal raceme (local open raceme density) was recorded after the field observations. We selected a 1.5 m radius because it reflected the local flowering condition of the focal raceme (variation in raceme density within this radius was small). When the focal raceme was at the edge of a patch, empty areas were included in the 1.5 m radius as it reflected the local flowering conditions. We used raceme density, instead of flower density, because the mean number of open flowers per raceme had little variation among patches.

Using the videos of the field observations, we recorded pollinator visits to racemes (raceme visits) and flowers (flower visits), and visited flower ratio. To count raceme visits, for each raceme observed, we counted the number of effective visits during the 3 h of observation. We regarded a visit as effective if a pollinator entered a flower and its body touched the anthers. Pollinator behaviour approaching flowers but not touching the anthers were not counted, and a revisit to a probed flower within a single bout was counted as a new visit. To count flower visits, for each flower within the raceme observed, we counted the number of effective visits during the 3 h of observation. Here, we counted multiple visits to a flower when pollinators re-visited the same flower during single visits to their racemes. To obtain the visited flower ratio, we recorded the numbers of visited and not visited open flowers in each raceme for each pollinator visit to determine the proportion of visited flowers to all open flowers in the raceme for each pollinator visit. Note that the visited flower ratio could be related to decision making about leaving racemes because this measurement involves the number of flowers not visited.

### Seed development

We marked all the flowers observed, including both pollinated and not pollinated ones, after pollinator observation. Seeds developed to mature size 2–3 weeks after pollination, and we could classify seeds as matured by their sizes. When seeds matured, we counted the number of seeds developed for each flower of each raceme studied to examine the effects of pollinator visits on seed production. We measured the basal stem diameter of the ramet of each raceme, which was used as an index of the ramet resource status in many previous studies e.g., ^50–53^ and possibly affects seed production. Also, individuals of *S. nipponica* having larger diameters tend to have longer stems (Fig. S2). We determined the seed development ratio (the number of seeds developed/4, where 4 is the number of ovules) for each flower of each raceme.

### Data analysis

For all statistical analyses, we applied a generalized linear mixed model (GLMM) using R version 4.3.1 statistical software^54^ with the *lme4* package^55^. Before modelling, patch size and distance from the nearest patch were log-transformed to weaken collinearity among explanatory variables (Pearson’s correlation coefficients between patch size and distance from the nearest patch before and after log-transformation were 0.79 and 0.48, respectively). After log-transformation, all values of the variance inflation factor (VIF) for all analyses were less than 2.6 (Supplementary Table S2) and hence the collinearity should not have large effects on the analyses. Because there were no evident differences in the measured variables between the north and south patches (Fig. 1, Supplementary Fig. S1), we analysed the data obtained from the north and south patches together.

To examine factors affecting pollinator preferences in visiting racemes, we developed a model in which number of raceme visits (number of pollinator visits to a raceme during its observation) was the response variable. The explanatory variables of the model were floral display size, local open raceme density, patch size, and distance from the nearest patch. Because these explanatory variables might have interactive effects on the response variable, two-way interaction terms of combinations of the four explanatory variables were also included. In addition, to consider yearly differences in the response variable and in the relationships between the explanatory variables including the two-way interactions and the response variable, observation year and interaction terms between year and other explanatory variables were also included. For the patch-level explanatory variables (patch size and distance to the nearest patch), the patch value was assigned to all racemes in the patch. For the number of open flowers, the initial value of each focal raceme at the beginning of pollinator observation was used. For this model, the Poisson error distribution with the log-link function was used.

To examine factors affecting pollinator preferences in visiting flowers, we developed a model in which the number of flower visits (number of pollinator visits to a flower including revisits during its observation) was the response variable. This model used the same set of explanatory variables and interactions as the model above. For the patch-level explanatory variables (patch size and distance from the nearest patch), the patch value was assigned to all flowers in the patch. For the local open raceme density, the value of each focal raceme was assigned for all flowers within it. For the number of open flowers, the value of the raceme bearing the focal flower when the first pollinator visit was observed was used. Note that if no pollinator visits were observed for a flower, the initial number of open flowers of the raceme bearing the flower was used. For the error distribution, the Poisson error distribution with the log-link function was used.

To examine factors affecting pollinator decisions about staying on a raceme or leaving it, we developed models in which the visited flower ratio among the open flowers in a raceme in one pollinator bout was the response variable. For this analysis, the data obtained in the first three bouts observed was used for each raceme and later bouts were discarded to avoid possible effects of reduction in nectar amount. The same explanatory variables used in the models described above were also used for this model. Similarly, the patch-level explanatory variables were assigned to all racemes in the patch. For the number of open flowers in the visited flower ratio model, the value of the focal raceme at the time of visitation was used. For the error distribution, the binomial error distribution with the logit-link function was used.

To avoid estimation failure and to compare relative effects of each explanatory variable, all explanatory variables (display size, local open raceme density, patch size, and distance from the nearest patch) were standardized to have mean = 0 and standard deviation = 1 using the *scale* function. The standardization was done for each dataset for each analysis after assigning patch-level and raceme-level variables to racemes and flowers so that the standardization did not affect the results of the analyses.

To control the potential dependencies in pollinator behaviour recorded within the same observation days, patches, ramets and racemes, all models included observation day, patch, ramet and raceme as random effects. However, models including all the random effects occasionally produced singular fits. Because there was no consensus about how to deal with singularity^55^ (please see the documentation of ‘isSingular’ in the lme4 package), we followed the method proposed by Matuschek et al.^56^ and selected random terms using step-wise likelihood ratio tests with α = 0.2 to avoid singularity. Using the resultant models, the significance of the explanatory variables was tested by type II Wald χ^2^ tests using the *Anova* function in the *car* package^57^ because the significance of estimated coefficients using *z* statistics produced by the *summary* function depends on correspondence of the dummy variable for the year term.

To find which aspects of pollinator behaviour were important for seed production, a GLMM testing relationship between pollinator behaviour and seed development was implemented. The response variable of the model was the seed development ratio of a flower, and the explanatory variables were the number of raceme visits, the average visited flower ratio of the raceme where the flower was located, and the number of flower visits. In addition, basal stem diameter of the ramet of each raceme was included as an explanatory variable which might represent the resource status of individual plants. Because seed development and the relationships between the explanatory variables and seed development could be different between 2019 and 2020, observation year and two-way interaction terms between each explanatory variable and year were also used as explanatory variables. All explanatory variables were standardized using the same method as the other models. To control for potential similarities in seed set within the same patches, ramets, or racemes, the model included patch, ramet, and raceme as random effects. Also, to control potential overdispersion in the response variable, flower was also included as a random effect. After implementing the model with all the random effects, the same method of model selection and hypothesis testing used in the other models was applied.

## Results

### Pollinators observed

The most frequent visitor was *Bombus diversus* Smith, the only species effective for pollination. This bumblebee visited several flowers in single racemes and represented the entirety of bumblebee visitations for observed racemes. These pollinator conditions were consistent for both years.

### Number of raceme visits

We recorded 419 and 575 pollinator visits to racemes in 2019 and 2020, respectively. The mean frequency of visits per raceme in 2020 was about twice as high as in 2019; 3.42 ± 2.45 and 10.26 ± 5.09 visits/3 h (mean ± SD) in 2019 and 2020, respectively.

**Figure 2:**
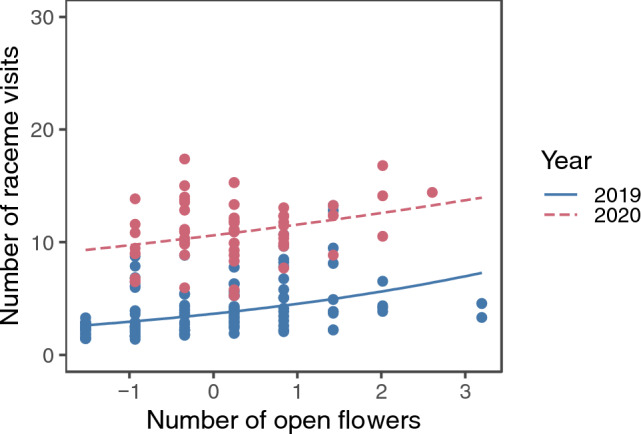
The predicted relationship between the number of raceme visits and the standardized number of open flowers in a raceme in 2019 and 2020. The lines represent the predicted relationships, and the points represents partial residuals. Values of other explanatory variables in the model (i.e., local open raceme density, patch size, and distance from the nearest patch) were fixed at their means when calculating the predictions. Also, because we found significant interaction between year and local raceme density (Appendix S4), the difference between the 2 years could depend on local raceme density.

**Figure 3:**
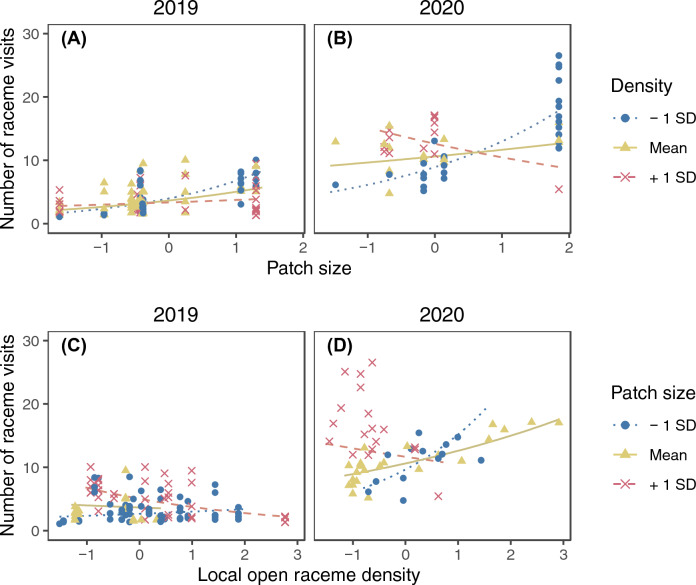
The interactive effect of patch size and local raceme density on the number of raceme visits in 2019 (**A** and **C**) and 2020 (**B** and **D**). Lines represent predicted relationships between the standardized patch size (**A** and **B**) or local raceme density (**C** and **D**) and the number of raceme visits when another explanatory variable is mean – 1 SD (dotted), mean (solid), and mean + 1 SD (dashed). The points represent partial residuals and their colours and symbols show the range of another explanatory variable (circle: $x \leqq -0.5$; triangle: $-0.5<x<0.5$; cross: $x\geqq 0.5$). Lines are drawn for the same range of another explanatory variable to avoid extrapolation. Values of other explanatory variables which are not shown in the panels were fixed at their means when calculating the predictions. Note that log-transformation was applied for patch size before standardization.

**Figure 4:**
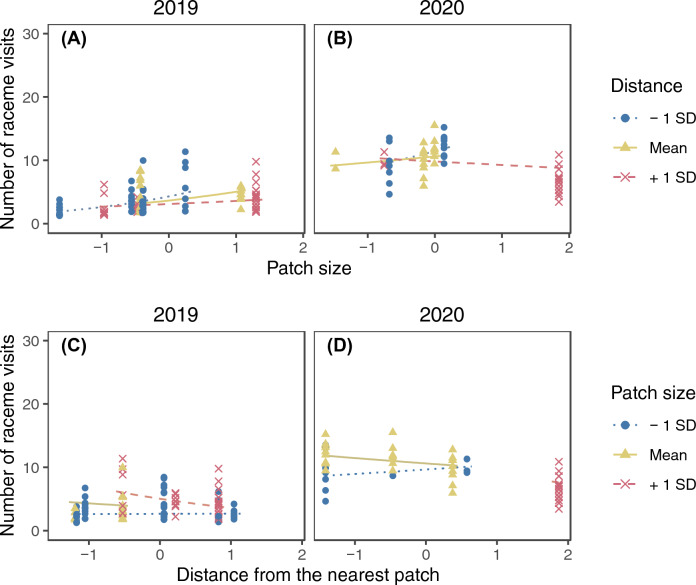
The interactive effect of patch size and distance from the nearest patch on the number of raceme visits in 2019 (**A** and **C**) and 2020 (**B** and **D**). Lines represent predicted relationships between the standardized patch size (**A** and **B**) or distance from the nearest patch (**C** and **D**) and the number of raceme visits when another explanatory variable is mean−1 SD (dotted), mean (solid), and mean + 1 SD (dashed). The points represent partial residuals and their colours and symbols show the range of another explanatory variable (circle: $x \leqq -0.5$; triangle: $-0.5<x<0.5$; cross: $x\geqq 0.5$). Lines are drawn for the same range of another explanatory variable to avoid extrapolation. Log-transformation was applied for patch size and distance from the nearest patch before standardization.

The model selection procedure kept ramet identity as the sole random factor for the final model of the raceme visits (SD of the random factor = 0.245). The number of raceme visits increased with increasing number of open flowers in a raceme and was higher in 2020 (Fig. 2; Supplementary Table S3). Significant interactions were found between the local open raceme density and patch size, patch size and distance from the nearest patch, and the local open raceme density and year (Supplementary Table S3). The patch size and open raceme density had interactive effects on raceme visits. In both years, the number of raceme visits was higher in larger patches only when the local open raceme density was low and in 2020 the trend was reversed if local raceme density was high (Fig. 3A and B). On the other hand, local raceme density had positive effects on the number of raceme visits in small patches whereas it had negative effects in large patches in both years (Fig. 3C and D). The patch size and distance from the nearest patch had interactive effects on raceme visits (Fig. 4). The number of raceme visits was higher in larger patches only when the nearest patch was closer (Fig. 4A and B). On the other hand, distance from the nearest patch had negative effects for large patches (Fig. 4C and D).

The observed number of raceme visits showed a wider range of responses depending on patch size and local open raceme density (Fig. 3; predicted lines ranged from ca. 2 to 8 in 2019 and from ca. 8 to 18 in 2020) than on the number of open flowers (Fig. 2; it ranged from ca. 3 to 6 in 2019 and from ca. 10 to 13 in 2020) and the interaction between distance from the nearest patch and patch size (Fig. 4; it ranged from ca. 2 to 5 in 2019 and from ca. 8.5 to 10 in 2020).

### Number of flower visits

**Figure 5:**
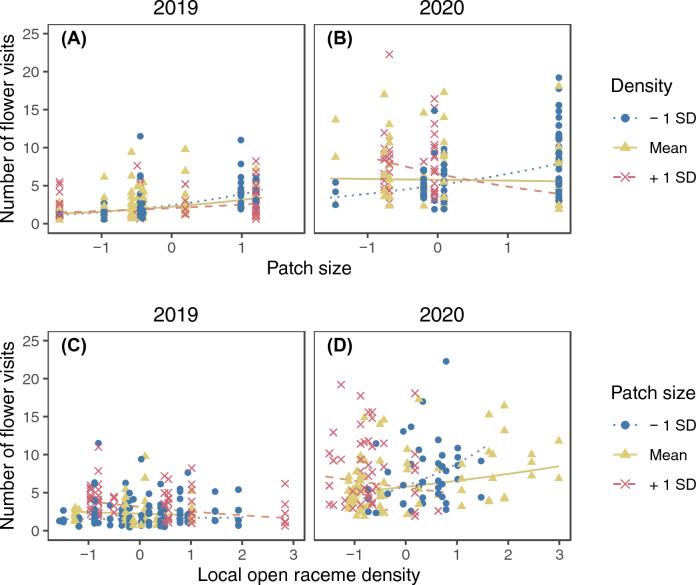
The interactive effect of patch size and local raceme density on the number of flower visits in 2019 (**A** and **C**) and 2020 (**B** and **D**). Lines represent predicted relationships between the standardized patch size (**A** and **B**) or local raceme density (**C** and **D**) and the number of flower visits when another explanatory variable is mean −1 SD (dotted), mean (solid), and mean + 1 SD (dashed). The points represent partial residuals and their colours and symbols show the range of another explanatory variable (circle: $x \leqq -0.5$; triangle: $-0.5<x<0.5$; cross: $x\geqq 0.5$). Lines are drawn for the same range of another explanatory variable to avoid extrapolation. Log-transformation was applied for patch size before standardization.

**Figure 6:**
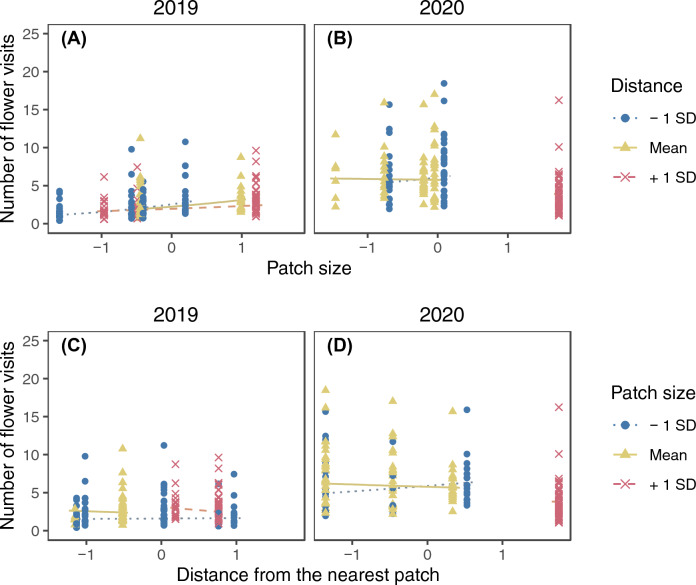
The interactive effect of patch size and distance from the nearest patch on the number of flower visits in 2019 (**A** and **C**) and 2020 (**B** and **D**). Lines represent predicted relationships between the standardized patch size (**A** and **B**) or distance from the nearest patch (**C** and **D**) and the number of flower visits when another explanatory variable is mean −1 SD (dotted), mean (solid), and mean + 1 SD (dashed). The points represent partial residuals and their colours and symbols show the range of another explanatory variable (circle: $x \leqq -0.5$; triangle: $-0.5<x<0.5$; cross: $x\geqq 0.5$). Lines are drawn for the same range of another explanatory variable to avoid extrapolation. Log-transformation was applied for patch size and distance from the nearest patch before standardization.

The model selection procedure kept ramet identity as the sole random factor for the final model of the flower visits (SD of the random factor = 0.370). For the number of flower visits, significant interactions were found between local open raceme density and year, patch size and year, local open raceme density and patch size, and patch size and distance from the nearest patch (Supplementary Table S4). In both years, the number of flower visits was higher in larger patches but only when local open raceme density was low (Fig. 5A and B). In 2020, patch size had a negative effect on number of flower visits when local raceme density was high (Fig. 5B). Local open raceme density generally had a negative effect on the number of flower visits in 2019 (Fig. 5C). In contrast, it had negative effects in large patches whereas it had positive effects in smaller patches in 2020 (Fig. 5D). In addition, the number of flower visits was higher in larger patches only if they were closer to other patches in both years (Fig. 6A and B). In both years, flowers in distant patches received smaller numbers of visits in large patches (Fig. 6C and D).

### Visited flower ratio among the open flowers in a raceme

**Figure 7:**
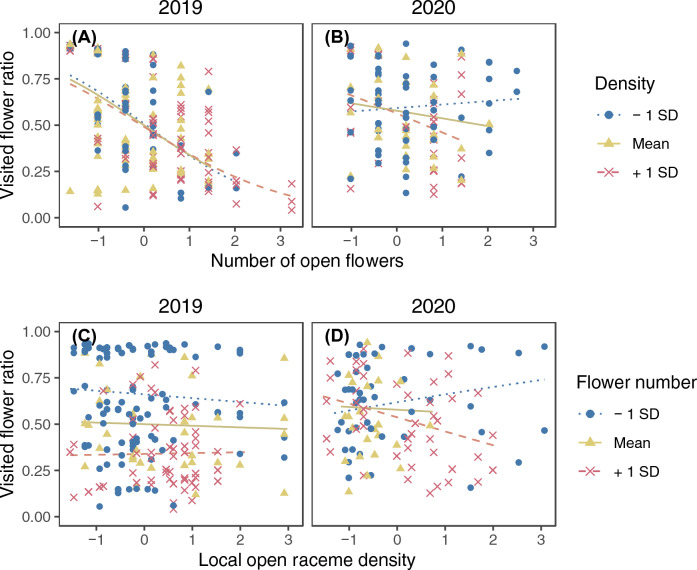
The interactive effect of the number of open flowers and local open raceme density on the visited flower ratio in 2019 (**A** and **C**) and 2020 (**B** and **D**). Lines represent predicted relationships between the standardized number of open flowers (**A** and **B**) or local open raceme density (**C** and **D**) and the visited flower ratio when another explanatory variable is mean − 1 SD (dotted), mean (solid), and mean + 1 SD (dashed). The points represent partial residuals and their colours and symbols show the range of another explanatory variable (circle: $x \leqq -0.5$; triangle: $-0.5<x<0.5$; cross: $x\geqq 0.5$). Lines are drawn for the same range of another explanatory variable to avoid extrapolation.

**Figure 8:**
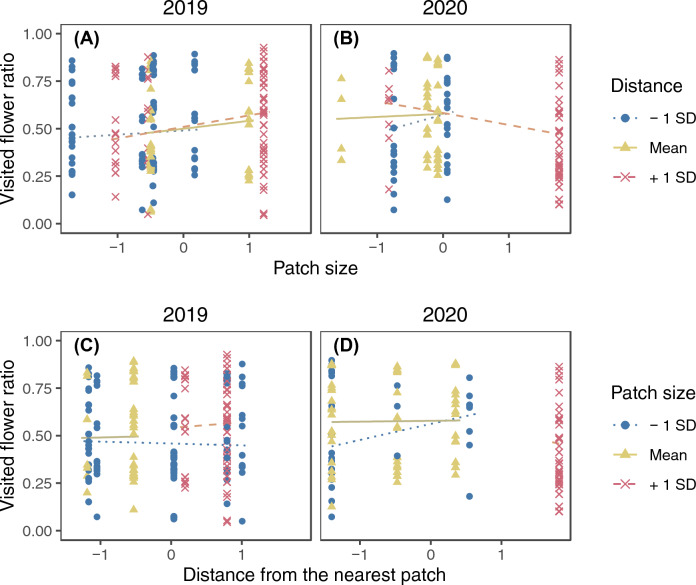
The interactive effect of patch size and distance from the nearest patch on the visited flower ratio in 2019 (**A** and **C**) and 2020 (**B** and **D**). Lines represent predicted relationships between the standardized number of patch size (**A** and **B**) or distance from the nearest patch (**C** and **D**) and the visited flower ratio when another explanatory variable is mean − 1 SD (dotted), mean (solid), and mean + 1 SD (dashed). The points represent partial residuals and their colours and symbols show the range of another explanatory variable (circle: $x \leqq -0.5$; triangle: $-0.5<x<0.5$; cross: $x\geqq 0.5$). Lines are drawn for the same range of another explanatory variable to avoid extrapolation. Log-transformation was applied for patch size and distance from the nearest patch before standardization.

The model selection procedure kept raceme identity as the sole random factor for the final model of the visited flower ratio (SD of the random factor = 0.319). The number of flowers effectively visited in a raceme during a single visit (not counting revisits to the same flowers) was 1.54 ± 0.93 (mean ± SD) in 2019 and 1.93 ± 1.30 in 2020. Significant three-way interactions on their effects on the visited flower ratio were found among the number of open flowers, local open raceme density, and year, and among patch size, distance from the nearest patch, and year (Supplementary Table S5). The visited flower ratio was higher in racemes with smaller display sizes whereas local open raceme density had a negligible effect in 2019, and the interaction between display size and raceme density was not strong (Fig. 7A and C). On the other hand, the visited flower ratio was higher in racemes with smaller display sizes if the racemes had higher local open raceme density whereas it was slightly higher in racemes with larger display sizes if the racemes had lower raceme density in 2020 (Fig. 7B). Local raceme density had a positive effect on the visited flower ratio for smaller racemes whereas the opposite relationship was observed for larger racemes (Fig. 7D). Distance from the nearest patch and patch size did not have strong effects on the visited flower ratio in 2019 (Fig. 8A and C) whereas in 2020 it was higher in larger patches that were closer to other patches and smaller patches that were distant from other patches (Fig. 8B and D).

The observed visited flower ratio showed wider responses depending on the number of open flowers and local open raceme density (estimated slope in Fig. 7; it ranged from ca. 0.15 to 0.75 in 2019 and from ca. 0.3 to 0.75 in 2020) than on patch size and distance (Fig. 8; it ranged from ca. 0.45 to 0.58 in 2019 and from ca. 0.35 to 0.6 in 2020). In particular, the response to the number of open flowers was very strong in 2019.

### Seed development ratio

**Figure 9:**
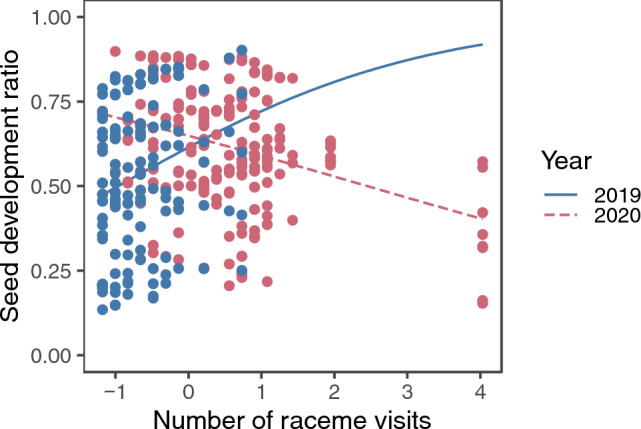
The predicted relationship between the standardized number of raceme visits and seed development ratio in 2019 and 2020. The lines represent the predicted relationships for the two years, and the points represent partial residuals.

The model selection procedure kept ramet and flower identities as the random factors for the final model of the seed development ratio (SD of the random factor; ramet ID: SD = 0.783; flower ID: SD = 0.570). Among the observed aspects of pollinator behaviour, the interaction between the number of raceme visits and year had the strongest effect on the seed development ratio (Supplementary Tables S6 and S7), though the *p* value was slightly higher than 0.05; the seed development ratio tended to increase with the number of raceme visits in 2019 but tended to decrease with it in 2020 (Fig. 9).

## Discussion

Pollinator behaviour was affected by the floral display size and the local flowering conditions including the local raceme density, patch size, and distance from the nearest patch. We found several consistent effects of the local flowering conditions in both years, though there were differences between the 2 years.

### Decision making using multiple signals

#### Raceme visits

The number of raceme visits was affected by both display size (Fig. 2) and interactions between patch level variables (Figs. 3 and 4). Patch size and local open raceme density had larger effects on raceme visits than the number of open flowers (compare changes along the variables in Fig. 3 with Fig. 2). This suggests that the decision making of pollinators about visiting racemes relies more on patch size and local density than on display size. Maybe this is because the benefit of an entire patch (patch size) or the cost of movement among racemes (local density) have larger effects on the foraging efficiency of pollinators than the benefit of individual racemes (raceme size). Also, no significant interaction between display size and patch level variables was detected (Supplementary Table S3). This suggests that pollinators rely on the display size independent of the other patch level signals; maybe visiting racemes having large display sizes improves foraging efficiency of pollinators in any patch level conditions.

The number of raceme visits increased with increasing number of open flowers in the raceme (Fig. 2), consistent with many previous studies^1,4–9^. However, the local flowering conditions also affected the number of raceme visits; one of the consistent results in both years was that the effect of local raceme density on raceme visits depended on patch size: it was higher in the locations with lower local raceme density in larger patches (Fig. 3). The local raceme density may be important in large patches compared to small patches because sub-structures of raceme distributions within patches may be more important in large patches, where the cost of within-patch movement is large, than in small patches. Mustajärvi et al.^28^ also reported that pollinator visits per plant were higher in sparse populations. However, many other studies^6,7,12,13,27^ reported that pollinator visits per plant were high in dense areas. Perhaps the interactive effect of patch size and local raceme density we showed can explain the difference in the density dependence of pollinator visits among these studies.

The number of raceme visits was also affected by the interaction between patch size and distance from the nearest patch though the effect was smaller than other explanatory variables (Fig. 4): it was higher in larger patches that are closer to other patches in both years (Fig. 4). This might be because patches within a certain distance are subjects for patch selection; for pollinators that have left a patch, large patches soon found are more attractive compared to small patches soon found, resulting in higher raceme visits in nearby, large patches. Thus, such decisions may be in agreement with previous studies showing frequent visits of pollinators in larger^2,15^ or closer^2^ patches. Thus, pollinators may select patches providing higher benefits with lower search costs. On the other hand, no obvious trend was found in the relationship between the number of raceme visits and patch size in patches distant from others (Fig. 4A and B). This suggests that pollinators do not selectively visit patches after long trips from previously visited patches because further trips are costly and there is no assurance of finding better patches.

#### Flower visits

The number of flower visits showed a similar response to the local flowering conditions as the number of raceme visits; the consistent results in both years were that the number of flower visits was higher in locations with lower local raceme density in larger patches (Fig. 5), and in larger patches that were closer to other patches (Fig. 6). This may be simply because pollinators visit more flowers while frequently visiting racemes.

According to the IFD, the proportions of foraging animals will equal the rates of resource production^58^. For pollinators, increasing number and decreasing proportion of visited flowers with increasing size of flower clusters often bring equal frequency of pollinator visits to each flower irrespective of cluster size and produce IFD of pollinators^2,19^. However, our results showed scale-dependent deviation from the IFD; although there was no relationship between raceme display size and flower visits, flower visits were higher in larger patches when the focal patches were close to other patches (Fig. 6). This might be because the cost of movement between clusters of flowers and incomplete knowledge of resource distribution, which are neglected in the original IFD model and may increase with distance, are more significant at larger spatial scales. On the other hand, the observed pattern was different from the prediction of the revised IFD model incorporating suboptimal movement due to incomplete discriminability of benefit, distance-dependent cost of movement, and information uncertainty of resource distribution^20^. In the revised model, foragers were predicted to use smaller resource patches more frequently than the prediction of the original IFD model when forager movement is suboptimal. Under this condition, increasing information uncertainty shifted the distributions of foragers to more frequent use of smaller patches whereas increasing cost of movement shifted the distribution closer to the prediction of IFD. However, because bumblebees used flowers in larger patches more frequently (Fig. 6), the observed pattern did not match the prediction of the model. Another possibility which could explain the observed pattern would be differences in nectar production in different sized patches; larger patches could be located in favorable environmental conditions and flowers in larger patches could produce more nectar per flower. Although nectar production of *S. nipponica* does not depend on display size of inflorescences^3^, its dependence on patch size is unknown. Measuring nectar production in different sized patches may help to understand potential mechanisms underlying the scale-dependent deviation from IFD.

#### Visited flower ratio

The visited flower ratio may be based on decision making about staying on or leaving racemes, noting that this measurement involves the number of flowers not visited. This ratio was affected both by the number of open flowers and the studied patch conditions, but the number of open flowers and local open raceme density had stronger effects than patch size and distance (compare Fig. 7 and Fig. 8). This suggests that, in the decision making about leaving racemes, pollinators rely more strongly on the benefit of the current raceme and the cost of movement to adjacent racemes than on the total benefit of the current patch or the cost of arrival to the current patch. In addition, interactive effects on the visited flower ratio were found (Figs. 7 and 8, Supplementary Table S5). This suggests that pollinators determine when to leave racemes by balancing several factors relating the costs and benefits rather than relying on a single factor.

The visited flower ratio was higher in racemes with lower numbers of open flowers in 2019 (Fig. 7A). This result agrees with the prediction that, as pollinators have limited memory, pollinators forage in higher proportions of flowers in plants with smaller display sizes because of low probabilities of revisiting already-foraged flowers^21^. On the other hand, the interactive effect of the number of open flowers and local open raceme density was more evident in 2020: the visited flower ratio was slightly higher in racemes with larger numbers of open flowers in lower local open raceme densities, in contrast to the result in 2019, whereas it was higher in racemes with smaller numbers of open flowers in higher raceme densities, as in 2019 (Fig. 7B). These results in 2020 are consistent with a previous study reporting that the proportion visited (number of visited flowers in a bout/display size) was higher for larger display sizes in sparse patches and was higher for the smallest display sizes in dense patches^7^. The difference between the years might be due to pollinators being more abundant in 2020 compared to 2019. In 2020, there might have been more flowers previously foraged by other pollinators, reflecting increasing competition among pollinators. Under such conditions, pollinators may need to adjust their departure timing based on the number of open flowers in the current raceme relative to the cost of movement to adjacent racemes (i.e., local density). Note that because there were only two patches observed in both years, the difference between the years might be partly confounded by the spatial difference between the patches.

Although their effects were weaker than the other variables, patch sizes and distances also affected the visited flower ratio; it was higher in larger and distant patches in 2019 though the effect of distance alone was negligible among the observed patches (Fig. 8A and C), whereas it was higher both in smaller distant patches and in larger patches that were closer to other patches in 2020 (Fig. 8B and D). Note that the number of raceme visits also depended on patch size and distance from the nearest patch in a manner similar to the visited flower ratio; it was higher in larger patches slightly closer to other patches in 2019 whereas it was higher in smaller and distant patches and larger patches that are closer to other patches in 2020 (Fig. 4). Therefore, one possible explanation for the observed patterns is that pollinators adjusted their foraging behaviour in response to competition with other pollinators, which should be strong if the number of raceme visits is large. In patches with higher numbers of raceme visits, the benefit of movement to other racemes would decrease and the benefit of staying on the current raceme would increase because the probability of visiting already pollinated racemes would be higher. Therefore, the visited flower ratio of pollinators would be higher in patches with higher numbers of raceme visits.

### Effects of pollinator behaviour on seed production

The interaction between the number of raceme visits and year had the strongest effect on the seed development ratio, a measurement of effective pollen deposition (Supplementary Table S6); it tended to increase with the number of raceme visits in 2019 but tended to decrease with it in 2020 (Fig. 9). These contrasting results might be due to greater abundance of pollinators in 2020 than in 2019; it is possible that raceme visits enhanced seed production simply due to enhanced pollen deposition in 2019, whereas pollen deposition was saturated for seed production in 2020. However, it is unclear why raceme visits had negative effects on seed production in 2020. Miyake and Sakai^1^ showed that larger racemes of *S. nipponica* received higher numbers of raceme visits and geitonogamy, but outcrossing rates were still higher for larger racemes in the same study site. Therefore, the negative effect might not be caused by inbreeding depression by geitonogamy. Other factors such as resource depletion by nectar consumption and style damage by pollinators^59^ might be responsible for this negative effect.

## Conclusion

In this study, we found several interactive effects of display size and patch conditions on pollinator behaviour. Thus, in a natural population of flowering plants where patchy sub-structure causes uneven distribution of floral resources, pollinators use multiple signals representing the costs and benefits of raceme- and patch-level conditions and adjust their responses to a certain signal in association with other signals. Moreover, pollinators use different information signals when visiting and leaving racemes; pollinators may use patch-level information when visiting racemes, but raceme-level information when leaving racemes. These results emphasize the importance of evaluating the effects of multiple factors and their interactions on several aspects of pollination behaviour to fully understand optimal foraging strategies of pollinators in natural populations.

### Supplementary Information

Supplementary Figures.

Supplementary Tables.

## Data Availability

All data and programs are available at Dryad: 10.5061/dryad.0vt4b8h14
