# Supplementary material for: Effects of floral display size, local open raceme density, patch size, and distance between patches on pollinator behaviour in Salvia nipponica
Source: Sci Rep. 2024 Jan 10;14:967. doi: 10.1038/s41598-024-51327-w (PMC10781768; doi:10.1038/s41598-024-51327-w)
Supplement: Supplementary file 1 — Supplementary Figures. [file 41598_2024_51327_MOESM1_ESM.docx]

**Murakoshi et al. – Figure S1**

Box-whisker plots showing a) number of raceme visits, b) number of flower visits, c) visited flower ratio, d) display size (number of open flowers) and e) local open raceme density of racemes and bar plots showing f) patch size and g) distance from the nearest patch for each patch in each year. For the box plots, horizontal bars represent median values, boxes represent the first and third quartiles, whiskers represent ranges of data, and points represent outliers. For the display size of racemes which dropped flowers during the pollinator observation time, we showed the maximum number of flowers for each raceme.

(a)

(b)

(c)

(d)

(e)

(f)

(g)

**Murakoshi et al. – Figure S2**

Relationship between basal diameter and stem length of *S. nipponica*.
